# CTRP6‐mediated cardiac protection in heart failure via the AMPK/SIRT1/PGC‐1α signalling pathway

**DOI:** 10.1113/EP092036

**Published:** 2024-09-26

**Authors:** Tingting Fan, Ningjun Zhu, Mengli Li, Zhen Wang, Xianhe Lin

**Affiliations:** ^1^ Department of Cardiology The First Affiliated Hospital of Anhui Medical University Hefei Anhui China; ^2^ Department of Cardiology The Second Affiliated Hospital of Anhui Medical University Hefei Anhui China

**Keywords:** AMPK, CTRP6, heart failure, mitochondrion, PGC‐1α, SIRT1

## Abstract

Heart failure (HF) remains a significant global health concern with limited effective treatments available. C1q/TNF‐related protein 6 (*CTRP6*) is a member of the *CTRP* family analogous to adiponectin and its role in HF pathogenesis remains unclear. Here, we investigated the impact of *CTRP6* on HF progression. To mimic heart failure with reduced ejection fraction (HFrEF), we used isoproterenol injection in mice and administered adenovirus vectors expressing *CTRP6* (Ad‐*CTRP6*) via tail vein injection. We assessed cardiac function through echocardiography and histology. *CTRP6*'s effects on hypertrophy, fibrosis, apoptosis, oxidative stress and mitochondrial function were analysed. Downstream pathways (phosphorylated AMP‐activated protein kinase (p‐AMPK), sirtuin 1 (SIRT1) and peroxisome proliferator‐activated receptor γ coactivator 1‐α (PGC‐1α) were studied in heart tissues. In vitro, isoproterenol‐stimulated H9c2 cardiomyocytes were treated with *CTRP6* to examine viability, apoptosis, F‐actin and signalling proteins. Compound C was used to assess AMPK involvement. *CTRP6* expression was lower in the plasma of HF patients. In an isoproterenol‐induced HFrEF mouse model, adenovirus‐mediated overexpression of *CTRP6* ameliorated cardiac dysfunction and reduced cardiomyocyte apoptosis, oxidative stress, inflammation and myocardial injury markers. Mechanistically, *CTRP6* activation of the AMPK/SIRT1/PGC‐1α signalling pathway restored mitochondrial homeostasis, evidenced by reduced mitochondrial reactive oxygen species levels, increased ATP content, and enhanced mitochondrial complex I/III activities in cardiac tissues. In vitro studies using isoproterenol‐stimulated H9c2 cardiomyocytes corroborated these findings, demonstrating that *CTRP6* upregulation attenuated hypertrophy, apoptosis, oxidative stress and mitochondrial dysfunction. Furthermore, these effects were partially reversed by the AMPK inhibitor Compound C, implicating the involvement of the AMPK pathway in *CTRP6*‐mediated cardioprotection. *CTRP6* alleviates HF progression through the AMPK/SIRT1/PGC‐1α signalling pathway.

## Abstract (highlights)

- **What is the central question of this study?**
  What are the role and mechanisms of C1q/tumour necrosis factor‐related protein‐6 (*CTRP6*) in heart failure (HF) progression and its potential cardioprotective effects?
- **What is the main finding and its importance?**
*CTRP6* alleviates HF progression through the activation of the AMPK/SIRT1/PGC‐1α signalling pathway, reducing oxidative stress, inflammation and mitochondrial dysfunction. This may enable development of novel therapeutic strategies for HF management.

## 1 INTRODUCTION

Cardiovascular disease (CVD) remains the leading cause of mortality worldwide, claiming approximately one‐third of global deaths annually (Di Cesare et al., 2024; Lindstrom et al., 2022). Heart failure (HF), a common consequence of various cardiac disorders and accounts for a significant portion of CVD‐related fatalities including myocardial infarction, aortic valve stenosis, myocardial hypertrophy, and fibrosis (Snipelisky et al., 2019). HF is characterized by a clinical syndrome marked by dyspnoea, reduced exercise tolerance, fluid retention and malaise, stemming from impaired ventricular function due to structural or functional cardiac abnormalities (Tomasoni et al., 2019; Tsutsui, 2022). The pathogenesis of HF involves multifaceted processes, including cardiomyocyte loss, apoptosis, hypertrophy, fibrosis and ventricular remodelling, all of which contribute to diminished cardiac function (Bacmeister et al., 2019; Schwinger & Therapy, 2021; Wang et al., 2023). With advancements in medical science and technology, an increasing array of pharmacological and non‐pharmacological therapies have emerged for alleviating HF (MacDonald et al., 2023; Scarà et al., 2024). However, despite advancements in medical interventions, the incidence of HF remains alarmingly high, necessitating a deeper understanding of its molecular underpinnings and the identification of potential therapeutic targets to enhance patient outcomes.

Among the myriad proteins implicated in cardiovascular physiology and pathology, C1q/tumour necrosis factor‐related protein‐6 (*CTRP6*) emerges as a noteworthy candidate (Wong et al., 2008). Belonging to the adiponectin paralogue family, *CTRP6* exhibits widespread expression across various tissues (Zhang et al., 2017) and has been implicated in diverse physiological and pathological processes, including glucose metabolism, cardiometabolism, fibrosis, inflammation and autoimmunity (Xu et al., 2024; Zhang et al., 2022, 2023). Emerging evidence suggests a cardioprotective role for *CTRP6* in cardiovascular diseases. It exerts beneficial effects in mitigating ventricular remodelling following myocardial infarction. These effects include the inhibition of myofibroblast differentiation, suppression of extracellular matrix production and attenuation of cardiac fibroblast migration (Lei et al., 2015). Activation of the AMP‐activated protein kinase (AMPK) and Akt pathways contributes significantly to the protective mechanisms elicited by *CTRP6* against transforming growth factor β 1 (*TGFβ1*)‐induced fibrotic responses. Specifically, these pathways target the Smad‐independent myocardin‐related transcription factor‐A (RhoA/MRTF‐A) signalling pathway, thereby mediating the anti‐fibrotic effects of *CTRP6*. Additionally, *CTRP6* has been shown to mitigate myocardial ischaemia–reperfusion injury via modulating the Akt–glycogen synthase kinase 3 β (GSK)–*Nrf2* pathway (Liang et al., 2023), protect against adriamycin‐induced cardiotoxicity (Zheng et al., 2019) and inhibit post‐infarction cardiac fibrosis (Lei et al., 2015). Despite these promising findings, the specific involvement of *CTRP6* in HF remains largely unexplored.

In this study, we aimed to elucidate the role of *CTRP6* in HF pathogenesis using an isoproterenol (ISO)‐induced heart failure with reduced ejection fraction (HFrEF) mouse model and cell model. By investigating the biological functions and underlying mechanisms of *CTRP6* in HF development, we seek to advance our understanding of this complex disease and identify novel therapeutic avenues for its management.

## 2 METHODS

### 2.1 Ethical approval

Procedures involving human tissues were conducted under project authorization (YX2022‐110) from the Ethics Committee of The First Affiliated Hospital of Anhui Medical University. Procedures involving live animals were carried out under project authorization (AMU#ZX2022‐112) from the same institution. All experiments were performed in accordance with the guidelines and requirements established by the institution's animal welfare committee and conformed to the principles and regulations described previously (Grundy, 2015). Informed consent was obtained from all patients, and the study protocol was approved by the Ethics Committee of The First Affiliated Hospital of Anhui Medical University (Approval No. YX2022‐110). The study adhered to the ethical standards outlined in the *Declaration of Helsinki*.

### 2.2 Patients

We enrolled a total of 100 HF patients treated in the Department of Cardiology at The First Affiliated Hospital of Anhui Medical University from October 2022 to July 2023. Additionally, 100 healthy subjects who underwent physical examinations at our hospital during the same period were included as controls. Exclusion criteria comprised other clinical diseases, malignant tumours or a history of mental illness. Venous blood samples (5 mL) were collected from each patient on an empty stomach in the morning, followed by centrifugation to obtain serum, which was then stored at −80°C.

### 2.3 The establishment of the HFrEF mouse model

Adult male wild‐type (WT) mice (C57BL/6, 8 weeks old) were procured from The First Affiliated Hospital of Anhui Medical University. Mice were housed in a controlled environment with a constant temperature of 22°C, humidity of 50%, and a 12‐h light–dark cycle. Throughout the study period, mice had ad libitum access to food and water. Ethical approval for the experimental procedures was obtained from the Ethics Committee of The First Affiliated Hospital of Anhui Medical University. Following established protocols, HFrEF was induced in mice using ISO (Jin et al., 2020). Mice were divided into four groups (*n* = 15 per group): a control group, ISO group, ISO + Ad‐NC group and ISO + Ad‐*CTRP6* group. The model mice received subcutaneous injections of ISO (5 mg/kg, Sigma‐Aldrich, St Louis, MO, USA) twice daily for 2 weeks. Adenovirus vectors expressing *CTRP6* (Ad‐*CTRP6*) and negative control (Ad‐NC) were obtained from Hanbio Technology Ltd (Shanghai, China) and administered to mice via tail vein injection (1 × 10^10^ viral particles per mouse) for 3 consecutive days. Control mice received an equivalent volume of saline. Overexpression of *CTRP6* in control, ISO, ISO+Ad‐NC and ISO+Ad‐*CTRP6* mice was measured using RT‐qPCR.

### 2.4 Echocardiographic analysis

Mice were anaesthetized with 1% isoflurane via inhalation for echocardiographic analysis. Cardiac function was evaluated using the Vevo 2100 VisualSonics system (VisualSonics Inc., Toronto, Canada) with M‐mode imaging. Parameters including left ventricular internal dimension (LVID), left ventricular fractional shortening (LVFS) and left ventricular ejection fraction (LVEF) were measured. Subsequently, mice were euthanized using intraperitoneal injection of pentobarbital solution (60 mg/kg; Merck KGaA, Darmstadt, Germany). Death was confirmed by the cessation of the heartbeat and dilatation of the pupils. After confirming death, the thoracic cavity was carefully opened to expose the heart. The heart was then swiftly excised and rinsed in cold phosphate‐buffered saline (PBS) to remove excess blood. Cardiac tissues were collected for histological analysis, with half of the tissue being fixed in paraformaldehyde (PFA) for histology and the remaining portion stored at −80°C for further experimentation.

### 2.5 Haematoxylin and eosin staining

The heart tissues of mice were fixed in 4% PFA for 24 h, followed by dehydration in a gradient of alcohol and embedding in paraffin. Subsequently, the tissues were sectioned into 5 µm slices, deparaffinized and rehydrated. To measure the cross‐sectional area of cardiomyocytes, sections were stained with haematoxylin and eosin (HE). Images were captured using a microscope (Olympus, Tokyo, Japan) and analysed using ImageJ software.

### 2.6 Western blot

Tissues were homogenized using RIPA buffer, followed by centrifugation at 12,000 *g* for 30 min at 4°C to collect the supernatant. Similarly, cells were lysed with RIPA buffer. Proteins were separated by 10% SDS‐PAGE and transferred onto polyvinylidene difluoride membranes (Millipore, Burlington, MA, USA). After blocking, membranes were incubated overnight at 4°C with primary antibodies: collagen 1 (COL1‐ab6308, 1:800), fibronectin 1 (FN1; Sigma‐Aldrich, AV41490, 1:100), natriuretic peptide A (NPPA; Thermo Fisher Scientific, Waltham, MA, USA, 27426, 1:200), natriuretic peptide B (NPPB; Creative Diagnostics, Shirley, NY, USA, 7366RH, 1:300), Bax (Cell Signaling Technology, Danvers, MA, USA, 2772, 1:400), Bcl2 (Abcam, Waltham, MA, USA, 182858, 1:1000), AMPK (Abcam, 32047, 1:800), phosphorylated (p)‐AMPK (Cell Signaling Technology, 2531, 1:500), sirtuin 1 (SIRT1) (Proteintech, Rosemont, IL, USA, 13161‐1‐AP, 1:250), peroxisome proliferator‐activated receptor‐γ coactivator 1‐α (PGC‐1α) (Santa Cruz Biotechnology, Dallas, TX, USA, 518038, 1:50) and β‐actin (Abcam, 8227, 1:400). Subsequently, membranes were probed with horseradish peroxidase‐conjugated secondary antibodies (mouse, Thermo Fisher Scientific, A‐31430, 1:500; rabbit, Thermo Fisher Scientific, 31460, 1:700) for 2 h. Visualization was achieved using an enhanced chemiluminescence reagent (Beckman Colter, Brea, CA, USA).

### 2.7 Enzyme‐linked immunosorbent assay

The concentration of *CTRP6* in the serum of HF patients was determined using specific enzyme‐linked immunosorbent assay (ELISA) kits (Jiangcheng Bioengineering Institute, Nanjing, China). Concurrently, the concentrations of creatine kinase‐MB (CK‐MB) and lactate dehydrogenase (LDH) in the serum of mice were measured using specific ELISA kits (Jiangcheng Bioengineering Institute, Nanjing, China) following the manufacturer's instructions. Additionally, inflammatory‐related cytokines (tumour necrosis factor‐α (*TNF‐α*), interleukin (*IL*)‐*1β* and *IL‐6*) in the H9c2 cardiomyocytes of mice in each group were determined according to the instructions of the ELISA kits (Jiangcheng Bioengineering Institute). Triplicate measurements were performed for each sample to ensure data accuracy and reproducibility.

### 2.8 TUNEL assay

Terminal deoxynucleotidyl transferase dUTP nick end labelling (TUNEL) staining was performed using an in situ cell death detection kit (Roche, Basel, Switzerland). Heart tissue sections were dewaxed and rehydrated, followed by permeabilization with PBS containing 0.1% Triton X‐100. Subsequently, the sections were incubated with 50 µL of TUNEL reaction mixture for 1 h at 37°C. After incubation, the sections were counterstained with 4′,6‐diamidino‐2‐phenylindole (DAPI) to visualize the nuclei. Finally, fluorescence microscopy (Olympus) was used for observation.

### 2.9 Caspase‐3 activity detection

Caspase‐3 activity was assessed using the Ac‐DEVD‐AFC Caspase‐3 Fluorogenic substrate kit (BD Biosciences, San Jose, CA, USA) following the manufacturer's instructions. Heart tissues were lysed on ice, and the supernatant was collected after centrifugation. Protein quantification was performed, and 50 µg of proteins were added to the detection buffer containing 10 mM dithiothreitol and 50 µm DEVD‐AFC. The mixture was then incubated at room temperature for a specified period. Subsequently, fluorescence intensity was measured at 380/500 nm (excitation/emission) using a microplate reader (Molecular Devices, San Jose, CA, USA).

### 2.10 Detection of oxidative stress markers

Heart tissues were homogenized in 1 mL of 0.9% normal saline and then centrifuged at 12,000 *g* for 30 min at 4°C to obtain the supernatant. Biochemical kits from Beyotime (Shanghai, China) were used to determine the levels of superoxide dismutase (SOD), glutathione peroxidase (GSH‐Px) and malondialdehyde (MDA) in the tissue or cell supernatant according to the manufacturer's instructions.

### 2.11 ATP activity detection

An Enhanced ATP Assay Kit (Beyotime, Shanghai, China) was employed to determine ATP activity. Heart tissues were homogenized in 200 µL of ATP lysate buffer to ensure efficient extraction of ATP. The homogenate was then centrifuged to obtain the supernatant containing the extracted ATP. The ATP content in the supernatant was assessed following the manufacturer's instructions provided with the assay kit. Briefly, an aliquot of the supernatant was mixed with the ATP detection reagent provided in the kit. This mixture was then incubated for a specified period at room temperature to allow for the reaction between the luciferase enzyme and ATP. After the incubation period, the luminescence emitted due to the ATP‐luciferase reaction was measured using a luminometer or a plate reader equipped with a luminescence detection module. The luminescence intensity directly correlates with the amount of ATP present in the sample. To quantify ATP levels, a standard curve may be generated using ATP standards with known concentrations provided in the kit.

### 2.12 Detection of the activities of mitochondrial complexes I and III

To assess the activities of mitochondrial complexes I and III, mitochondria were isolated from tissue samples using the Mitochondria Isolation Kit (Beyotime, c3606) following standard protocols. Briefly, tissue homogenates were centrifuged to remove cellular debris, and the resulting supernatant containing mitochondria was collected and further processed to obtain purified mitochondrial fractions. The activities of mitochondrial complexes I and III were then evaluated using the Complex I or III Enzyme Activity Assay Kit (Abcam, 109721 and 287844) according to the provided user guides. For complex I activity assessment, isolated mitochondria were incubated with the assay buffer and substrate provided in the kit, and the ratio of NAD(+)/NADH was measured spectrophotometrically at 340 nm over a specified period. Similarly, complex III activity was assessed by monitoring the reduction of cytochrome *c* at 550 nm. Enzyme activity was calculated based on the rate of substrate conversion and normalized to protein concentration. The results were analysed statistically, and comparisons between experimental groups were made using appropriate tests.

### 2.13 Cell culture

10^6^ rat H9c2 cardiomyocytes (ATCC, Manassas, VA, USA) in a six‐well plate were cultured in Dulbecco's modified Eagle's medium (DMEM) supplemented with 10% fetal bovine serum (FBS), 100 U/mL streptomycin, and 100 U/mL penicillin at 37°C in a humidified atmosphere containing 5% CO_2_. To induce a HFrEF cell model, the cells were exposed to a 200 µM solution of ISO in the culture medium for 24 h as described previously (Lee et al., 2022). Subsequently, to investigate the function of the AMPK/SIRT1/PGC‐1α signalling pathway, cells were treated with 10 µM of the AMPK inhibitor Compound C (CC) for 2 h prior to further experimentation as described previously (Ye et al., 2018).

### 2.14 Cell transfection

To explore the impact of *CTRP6* on cellular responses to ISO and AMPK inhibition, we conducted cell transfection to establish overexpression models. The full‐length sequence of *CTRP6* was inserted into the pcDNA3.1 vector (Geenseed, Guangzhou, China) to create the *CTRP6*‐overexpressing construct, while an empty pcDNA3.1 vector served as a negative control (NC). Experimental groups comprised rat H9c2 cardiomyocytes subjected to ISO treatment followed by transfection with the empty vector (ISO+Oe‐NC), the *CTRP6*‐overexpressing construct (ISO+Oe‐*CTRP6*, ATGCTGCTGCTGCTGCTG), or the *CTRP6*‐overexpressing construct with pre‐treatment of the AMPK inhibitor Compound C (ISO+Oe‐*CTRP6*+CC). Transfection was executed using Lipofectamine 3000 reagent (Thermo Fisher Scientific) as per the manufacturer's instructions. Post‐transfection, cells underwent the designated treatments to elucidate the effects of *CTRP6* overexpression and AMPK inhibition on ISO‐induced cellular stress.

### 2.15 RT‐qPCR

To investigate gene expression levels of atrial natriuretic peptide (*ANP*), brain natriuretic peptide (*BNP*), and β‐myosin heavy chain (*β‐MHC*), total RNA was extracted from tissues and cells using TRIzol reagent (Thermo Fisher Scientific) following the manufacturer's instructions. RT‐qPCR was also conducted to examine *CTRP6* expression in mice and H9c2 cardiomyocytes. The extracted RNA was then reverse‐transcribed into cDNA using the Reverse Transcription Kit (Qiagen, Hilden, Germany). Reverse transcription was carried out using random hexamer primers, and the cDNA synthesis reaction was performed according to the manufacturer's protocol. Subsequently, quantitative real‐time PCR (RT‐qPCR) was performed using a SYBR Green PCR Kit (Takara, Shiga, Japan) on a real‐time PCR system. The reaction mixture consisted of a cDNA template, forward and reverse primers specific to each gene of interest, and SYBR Green Master Mix. The thermal cycling conditions included an initial denaturation step at 95°C for 10 min, followed by 40 cycles of denaturation at 95°C for 15 s, annealing at 60°C for 30 s, and extension at 72°C for 30 s. Relative gene expression levels were calculated using the $2^{-\Delta \Delta C_{t}}$ method, with glyceraldehyde‐3‐phosphate dehydrogenase (*GAPDH*) as the internal control. The forward and reverse primer sequences used in this study were as follows: *ANP* forward primer: 5′‐CGGAGAGGAAGCGGAAG‐3′, reverse primer: 5′‐AGGCTGATGAAGCCCTTGC‐3′; *BNP* forward primer: 5′‐TGGGCTCTCTGGAGAGCA‐3′, reverse primer: 5′‐GGGCTCCAGTGCCTTCTTC‐3′; *β‐MHC* forward primer: 5′‐AGCACCTTTGGAAGCTCCTC‐3′, reverse primer: 5′‐CAGTGGAGGAGGATGAGGAAG‐3′. *CTRP6* forward primer: 5′‐ATGGCCTACGACCTGTGAAC‐3′, reverse primer: 5′‐TCCAGGTGACAGTCGATGTC‐3′.

### 2.16 Cell counting kit‐8 assay

Cell viability was assessed using a Cell counting kit‐8 (CCK‐8) assay kit (Promoter Biotechnology Ltd, Nanjing, China). Rat H9c2 cardiomyocytes were seeded in a 96‐well plate at a density of 1 × 10^4^ cells per well and incubated for 24 h to allow for cell adhesion and growth. Following incubation, 10 µL of CCK‐8 solution was added to each well, and the plate was further incubated for 2 h at 37°C in a humidified atmosphere with 5% CO_2_. The absorbance of the formazan dye produced by viable cells was measured spectrophotometrically at 450 nm using a microplate reader (Molecular Devices).

### 2.17 Flow cytometry

Cell apoptosis was assessed using the fluorescein isothiocyanate (FITC)‐labeled Annexin V apoptosis detection kit (BD Biosciences). Rat H9c2 cardiomyocytes were harvested and resuspended in 1× binding buffer at a concentration of 1 × 10^6^ cells/mL. The cells were then stained with Annexin V–FITC and propidium iodide according to the manufacturer's instructions and incubated for 15 min in the dark. Annexin V‐FITC labels early apoptotic cells, while propidium iodide stains late apoptotic and necrotic cells. After staining, the ratio of apoptotic cells was determined using flow cytometry (FACS Calibur, BD Biosciences).

### 2.18 Phalloidin staining

To visualize actin filaments, H9c2 cardiomyocytes were fixed in 4% PFA and washed with PBS to remove excess fixative. Permeabilization of the cell membrane was achieved by treating the cells with 0.5% Triton X‐100 for 20 min. Subsequently, cells were stained with 5 µg/mL of phalloidin conjugated to a fluorophore (e.g., FITC) (Yeasen, Shanghai, China) for 30 min to label F‐actin. To counterstain the cell nuclei, DAPI was used. After washing with PBS to remove the unbound dye, the stained cells were visualized using a fluorescence microscope (Olympus) equipped with appropriate filter sets for FITC and DAPI fluorescence. Images were captured using a digital camera attached to the microscope.

### 2.19 Reactive oxygen species assay

Reactive oxygen species (ROS) levels in heart tissues were assessed using dihydroethidium (DHE) staining. Tissue sections were dewaxed and incubated in PBS containing 0.1% Triton X‐100 to permeabilize the cells. Subsequently, sections were treated with a DHE reaction solution for 1 h at 37°C to allow for the detection of ROS. After incubation, the sections were imaged using a fluorescence microscope (Olympus) equipped with appropriate filter sets. To assess mitochondrial ROS levels in tissues, sections were stained with MitoSOX Green (Thermo Fisher Scientific). MitoSOX Green was added to the tissue sections and incubated for 30 min at 37°C. Following incubation, the sections were washed, and mitochondrial ROS levels were detected using a fluorescence microscope (Olympus). In cell culture experiments, rat H9c2 cardiomyocytes were seeded in a 24‐well plate and incubated with 10 µM of 2′,7′‐dichlorofluorescin diacetate (DCFH‐DA) (Beyotime) at 37°C for 30 min in a dark environment to assess intracellular ROS levels. After incubation, the cells were washed, and fluorescence microscopy (Olympus) was used to visualize ROS levels.

### 2.20 JC‐1 assay for mitochondrial membrane potential

Mitochondrial membrane potential was assessed using the JC‐1 fluorescent probe (Beyotime). Rat H9c2 cardiomyocytes were seeded in six‐well plates and allowed to adhere for 24 h. Subsequently, cells were treated with superparamagnetic iron oxide nanoparticles (SPIO‐Serum) at a concentration of 100 µg Fe/mL for an additional 24 h to induce changes in mitochondrial membrane potential. For the detection of mitochondrial membrane potential, cells were incubated with 2 µM of JC‐1 probe (Thermo Fisher Scientific) for 15 min at 37°C. Following incubation, cells were washed with PBS to remove excess dye, and the images were captured using a laser scan confocal microscope (Carl Zeiss, Shanghai, China). JC‐1 forms aggregates in mitochondria with high membrane potential, emitting red fluorescence, whereas, in depolarized mitochondria, JC‐1 remains in the monomeric form and emits green fluorescence.

### 2.21 Mitochondrial permeability transition pore opening detection

Mitochondrial permeability transition pore (mPTP) opening was assessed using the Mitochondrial Permeability Transition Pore Detection Kit (Beyotime, C2009S) following the manufacturer's instructions. Briefly, rat H9c2 cardiomyocytes were treated with the appropriate experimental conditions, and mPTP opening was induced. Following induction, the extent of mPTP opening was determined by measuring the absorbance or fluorescence intensity using a plate reader (Molecular Devices) at an appropriate wavelength according to the assay protocol. Data obtained from the measurements were analysed to quantify the level of mPTP opening in response to the experimental treatments.

### 2.22 Statistical analysis

Data are presented as means ± standard deviation (SD) from three independent experiments. Statistical analysis was conducted using GraphPad Prism software (version 8.0, GraphPad Software Inc., San Diego, CA, USA). Group comparisons were performed using either one‐way analysis of variance (ANOVA) followed by post‐hoc Tukey's multiple comparison test or Student's *t*‐test, as appropriate. A *P*‐value less than 0.05 was considered statistically significant.

## 3 RESULTS

### 3.1 *CTRP6* ameliorates cardiac dysfunction in ISO‐induced HFrEF mice

**FIGURE 1:**
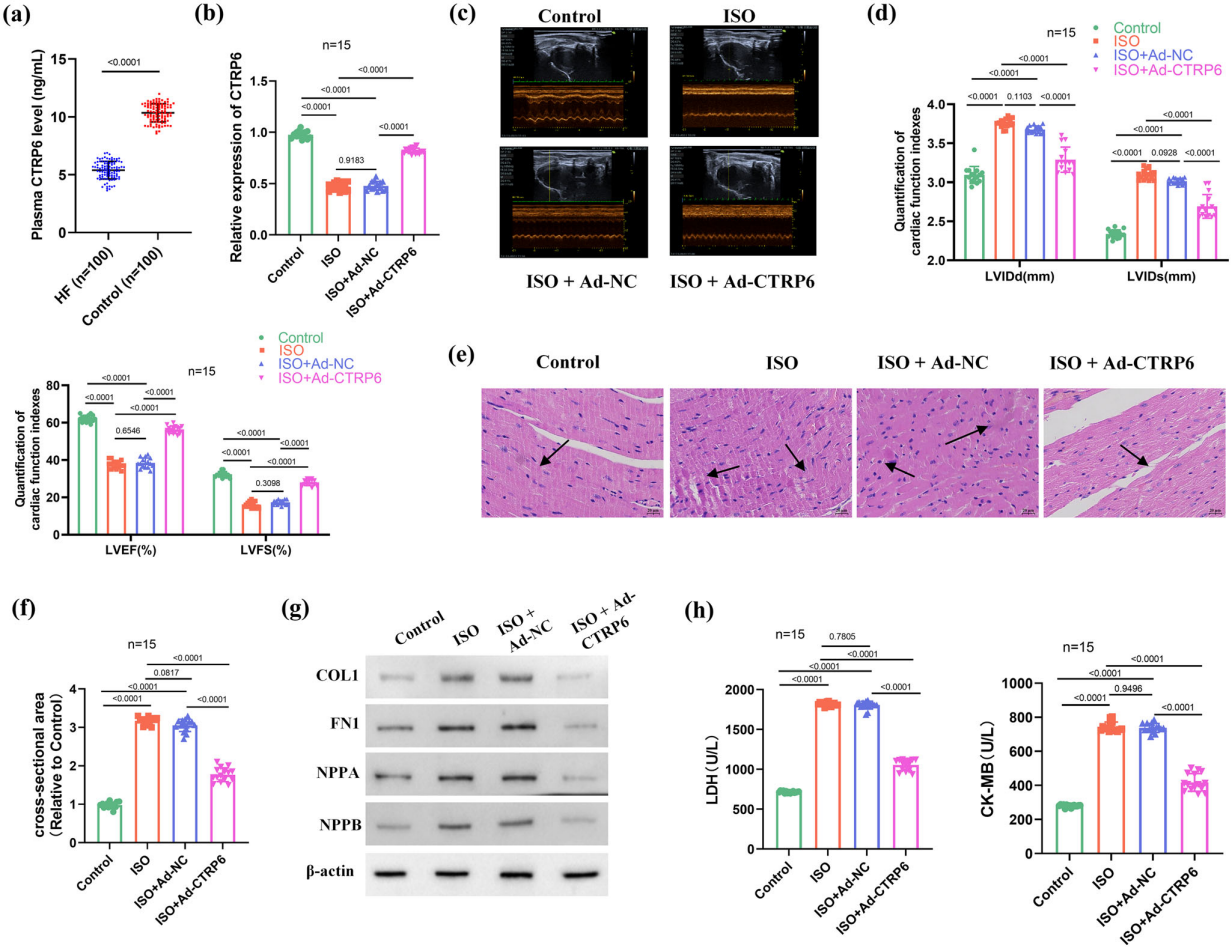
*CTRP6* ameliorates cardiac dysfunction in ISO‐induced HFrEF mice. (a) ELISA analysis of *CTRP6* levels in plasma samples from HF patients and controls (*n* = 100). (b) RT‐qPCR was used to investigate the *CTRP6* expression in the control, ISO, ISO+Ad‐NC and ISO+Ad‐*CTRP6* groups (*n* = 15). (c) Echocardiographic evaluation of mice in the control, ISO, ISO+Ad‐NC and ISO+Ad‐*CTRP6* groups (*n* = 15). (d) Quantification of echocardiographic parameters including LVIDd, LVIDs (upper right panel), LVEF and LVFS (middle left panel) in the control, ISO, ISO+Ad‐NC and ISO+Ad‐*CTRP6* groups (*n* = 15). (e) Representative images of HE‐stained heart sections showing histopathological changes in the control, ISO, ISO+Ad‐NC and ISO+Ad‐*CTRP6* groups. Scale bar: 20 µm. (f) Cardiomyocyte cross‐sectional area relative to the control for the ISO, ISO+Ad‐NC and ISO+Ad‐*CTRP6* groups (*n* = 15). (g) Western blot analysis of COL1, FN1, NPPA and NPPB protein levels in heart tissues of the control, ISO, ISO+Ad‐NC and ISO+Ad‐*CTRP6* groups (*n* = 15). (h) ELISA assessment of serum levels of CK‐MB and LDH in the control, ISO, ISO+Ad‐NC and ISO+Ad‐*CTRP6* groups (*n* = 15).

Initially, we assessed the expression of *CTRP6* in HF patients. ELISA results revealed a significant downregulation of *CTRP6* in the plasma of HF patients compared to controls (Figure 1a). To elucidate the regulatory role of *CTRP6* in HF development, we established an ISO‐induced HFrEF mouse model. *CTRP6* overexpression in HFrEF mice was achieved by transduction of adenoviral Ad‐*CTRP6* (Figure 1b). Echocardiography revealed that ISO induction notably decreased LVEF and LVFS, while increasing left ventricular internal diameter in diastole (LVIDd) and left ventricular internal diameter in systole (LVIDs). However, Ad‐*CTRP6* transduction effectively neutralized these alterations (Figure 1c,d). Subsequently, we investigated histopathological changes in the hearts of HFrEF mice using HE staining. Control mice exhibited morphologically normal and well‐arranged cardiomyocytes, whereas ISO induction resulted in significant cardiomyocyte hypertrophy and disorganization (Figure 1e). Additionally, the cardiomyocyte cross‐sectional area was markedly increased in the ISO group compared to controls (Figure 1f). Notably, *CTRP6* overexpression effectively mitigated these pathologic changes in cardiomyocytes and reduced the ISO‐induced increase in cross‐sectional area (Figure 1e,f). Given that cardiac fibrosis and hypertrophy are central processes in many cardiac dysfunction diseases, including HF, we further investigated the effects of *CTRP6* upregulation on markers of cardiac hypertrophy and fibrosis. Western blot analysis revealed that protein levels of collagen 1 (COL1), fibronectin 1 (FN1), natriuretic peptide A (NPPA) and natriuretic peptide B (NPPB) were significantly elevated in the ISO group compared to controls; however, *CTRP6* overexpression attenuated their expression levels (Figure 1g). Furthermore, we examined alterations in myocardial injury markers among the different groups. Lactate dehydrogenase (LDH) and creatine kinase‐MB (CK‐MB) levels were significantly elevated by ISO induction, but *CTRP6* overexpression abolished this increase (Figure 1h). Thus, our findings indicate that *CTRP6* ameliorates ISO‐induced cardiac dysfunction in HFrEF mice.

### 3.2 *CTRP6* reduces ISO‐induced cardiomyocyte apoptosis and oxidative stress in mice

**FIGURE 2:**
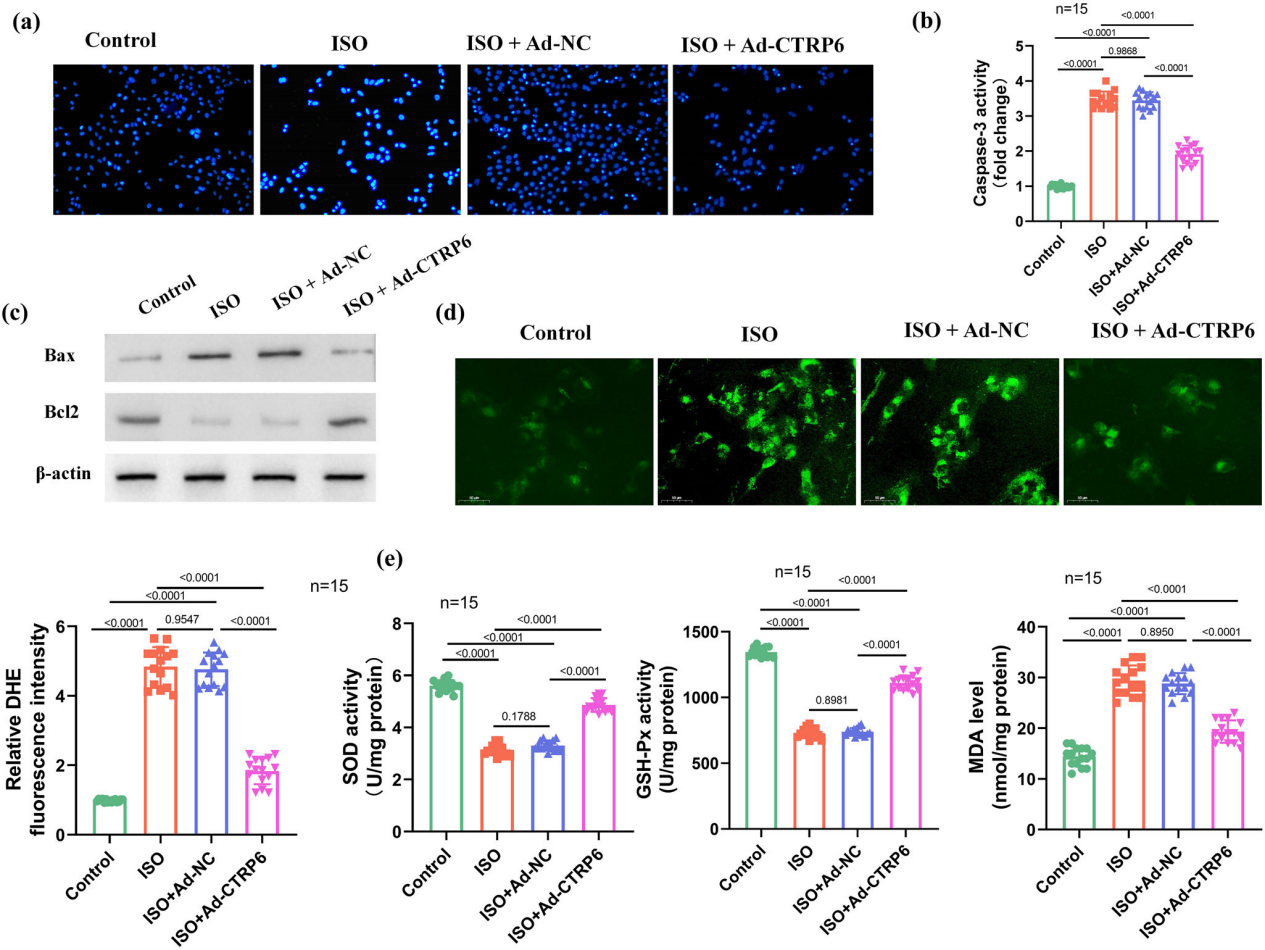
*CTRP6* reduces ISO‐induced cardiomyocyte apoptosis and OS in mice. (a) TUNEL assay was performed to evaluate the effect of *CTRP6* on apoptosis in heart tissues in the presence of ISO (*n* = 15). Scale bar = 100 µm. (b) Caspase‐3 activity in heart tissues of control, ISO, ISO+Ad‐NC and ISO+Ad‐*CTRP6* groups (*n* = 15) was measured to evaluate the effect of CTRP6 on apoptosis. (c) Western blot analysed the effect of *CTRP6* on the apoptosis‐related Bax and Bcl2 protein levels in heart tissues (*n* = 15). (d) Detection (middle right panel) and quantification (lower left panel) of ROS production using DHE fluorescence probes in the control, ISO, ISO+Ad‐NC and ISO+Ad‐*CTRP6* groups. Scale bar: 50 µm; *n* = 15 for the bar graph. (e) Measurement of SOD, GSH‐Px, and MDA concentrations in heart tissues of the control, ISO, ISO+Ad‐NC and ISO+Ad‐*CTRP6* groups (*n* = 15).

HF pathogenesis involves heightened oxidative stress (OS) response and cardiomyocyte apoptosis (Pang et al., 2023; Tsutsui et al., 2011). To investigate the potential therapeutic effects of CTRP6, we examined its impact on ISO‐induced cardiac injury in mice. Using TUNEL staining, we assessed the extent of apoptosis in cardiac tissues. ISO induction significantly increased apoptotic cell numbers, whereas *CTRP6* overexpression markedly attenuated this effect (Figure 2a). Consistent with TUNEL staining results, we observed elevated activity of caspase‐3, a key mediator of apoptosis in ISO‐treated hearts, which was substantially reduced upon *CTRP6* upregulation (Figure 2b) indicating that *CTRP6* suppresses ISO‐induced apoptosis by inhibiting caspase‐3 activation. Western blot analysis revealed alterations in the expression levels of Bax and Bcl2, pivotal regulators of apoptosis. ISO exposure upregulated Bax and downregulated Bcl2 expression, indicative of pro‐apoptotic signalling. Conversely, *CTRP6* overexpression mitigated these changes, restoring the balance between pro‐ and anti‐apoptotic proteins (Figure 2c). Additionally, using DHE fluorescent probes, we assessed ROS levels in cardiac tissues as excessive ROS production contributes to HF pathogenesis. ISO administration led to a significant increase in ROS production, whereas *CTRP6* overexpression attenuated this effect (Figure 2d). These findings suggest that *CTRP6* mitigates ISO‐induced OS in the heart. We further investigated the impact of *CTRP6* on antioxidant enzyme activities. ISO treatment resulted in decreased superoxide dismutase (SOD) and glutathione peroxidase (GSH‐Px) levels, accompanied by elevated malondialdehyde (MDA) content, indicative of oxidative damage. In contrast, *CTRP6* overexpression restored SOD and GSH‐Px levels while reducing MDA accumulation (Figure 2e) demonstrating the protective effects of *CTRP6* against ISO‐induced oxidative damage by enhancing antioxidant defences. Collectively, our findings indicate that *CTRP6* alleviates ISO‐induced cardiomyocyte apoptosis and OS in HFrEF mice.

### 3.3 *CTRP6* restores mitochondrial homeostasis in ISO‐induced mice

**FIGURE 3:**
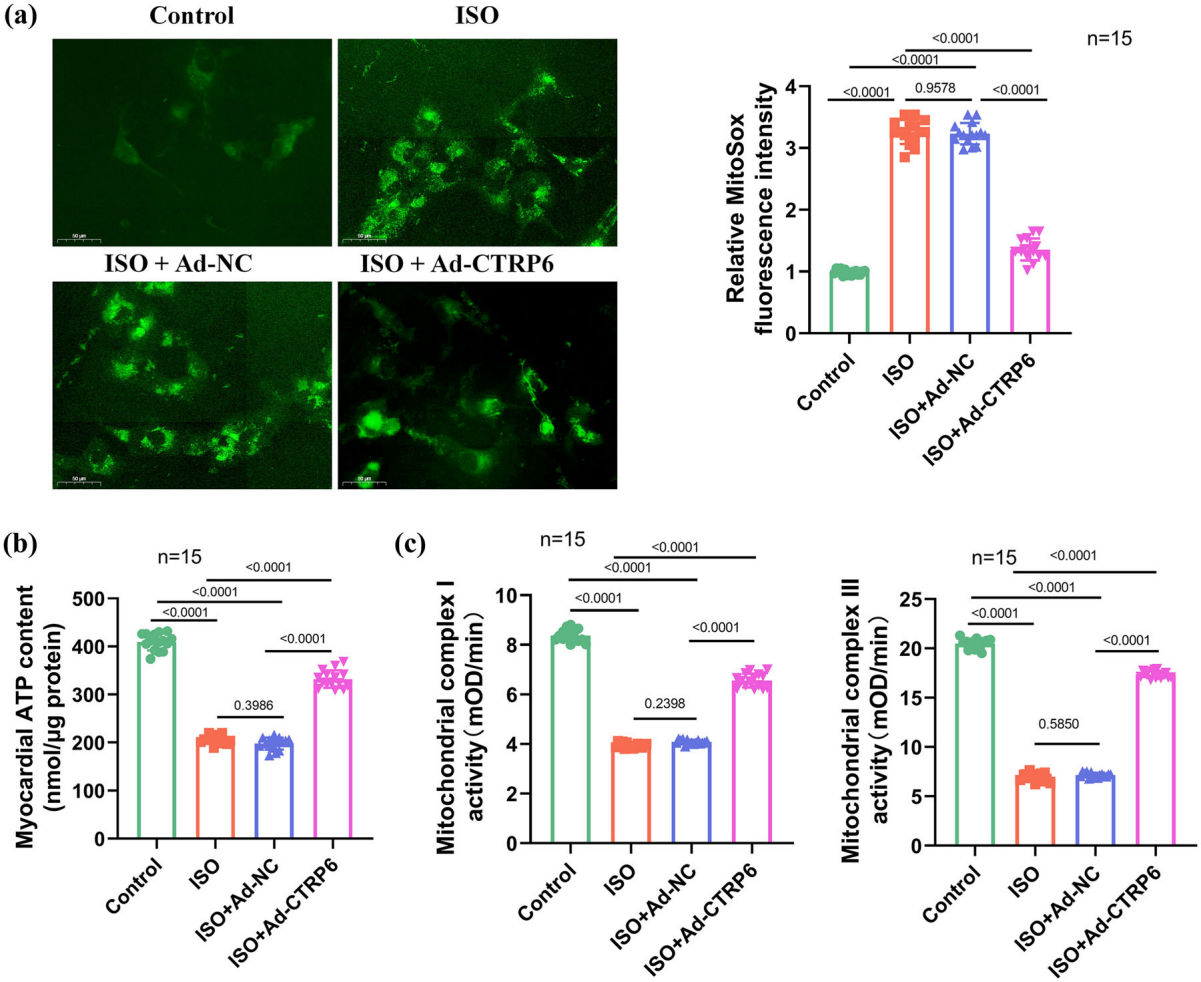
*CTRP6* restores mitochondrial homeostasis in ISO‐induced mice. (a) MitoSOX staining assay was performed to assess the effect of *CTRP6* on mitochondrial ROS levels in heart tissues. Scale bar: 50 µm; *n* = 15 for the bar graph. (b) Measurement of mitochondrial ATP content in heart tissues of the control, ISO, ISO+Ad‐NC and ISO+Ad‐*CTRP6* groups (*n* = 15) to evaluate the effect of *CTRP6* on mitochondrial function. (c) Assessment of mitochondrial complex I/III activities in heart tissues of the control, ISO, ISO+Ad‐NC and ISO+Ad‐*CTRP6* groups (*n* = 15) to evaluate the effect of *CTRP6* on mitochondrial respiratory chain function.

Mitochondria, known as the primary source of ROS, play a crucial role in cardiovascular diseases (Atici et al., 2023; Zhou & Tian, 2018). To assess the impact of *CTRP6* on mitochondrial function in ISO‐induced mice, we utilized a MitoSOX Green probe to measure mitochondrial ROS levels in cardiac tissues. We observed a substantial increase in green fluorescence intensity in the ISO group, indicative of elevated mitochondrial ROS production. However, in the ISO+Ad‐*CTRP6* group, we noted a significant decrease in fluorescence intensity, suggesting that *CTRP6* overexpression exerted an inhibitory effect on mitochondrial ROS production (Figure 3a). Furthermore, we evaluated ATP activity as a measure of mitochondrial function. Our results revealed that ATP activity, which was reduced following ISO induction, was restored in the presence of *CTRP6* overexpression (Figure 3b). Mitochondrial electron transport chain complexes I and III are essential for maintaining cardiomyocyte energy production (Li et al., 2019). We investigated the activities of these complexes in the heart tissues of mice subjected to ISO induction. Compared to controls, ISO induction led to a notable reduction in mitochondrial complex I and III activities. Remarkably, this detrimental effect was mitigated by *CTRP6* overexpression (Figure 3c). Collectively, our findings demonstrate that *CTRP6* restores mitochondrial function in ISO‐induced HFrEF mice.

### 3.4 *CTRP6* modulates the AMPK/SIRT1/PGC‐1α signalling pathway to attenuate ISO‐induced hypertrophy and apoptosis in H9c2 cardiomyocytes

**FIGURE 4:**
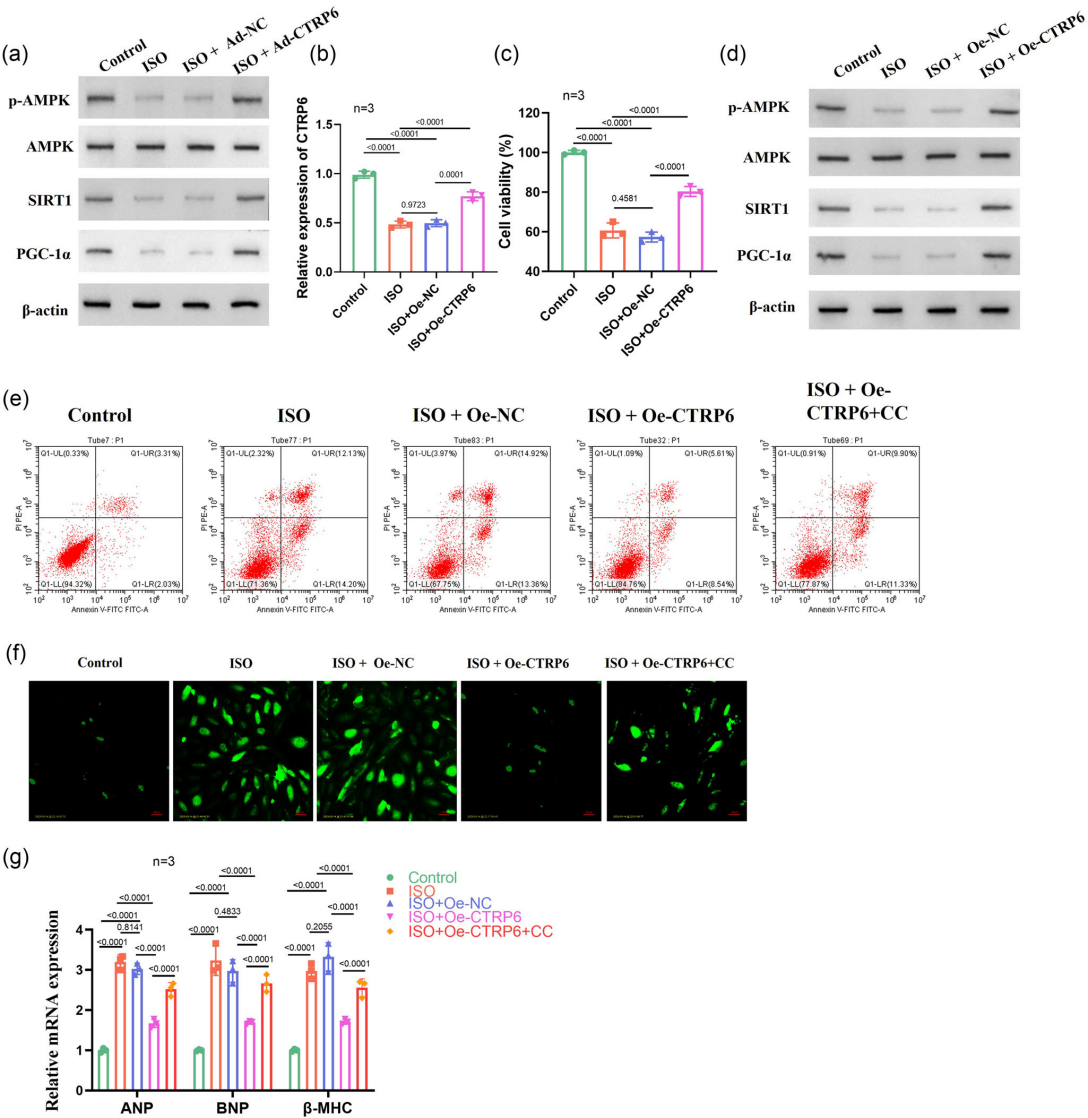
*CTRP6* modulates the AMPK/SIRT1/PGC‐1α signalling pathway and attenuates ISO‐induced hypertrophy and apoptosis in H9c2 cardiomyocytes. (a) Western blot analysis of phosphorylated AMPK (p‐AMPK), total AMPK, SIRT1 and PGC‐1α protein levels in heart tissues of the control, ISO, ISO+Ad‐NC and ISO+Ad‐*CTRP6* groups (*n* = 15). (b) RT‐qPCR detected *CTRP6* expression in H9c2 cardiomyocytes in the control, ISO, ISO+Oe‐NC and ISO+Oe‐*CTRP6* groups (*n* = 3). (c) CCK‐8 assay to evaluate the effect of *CTRP6* overexpression on the viability of H9c2 cardiomyocytes in the presence of ISO (*n* = 3). (d) Western blot analysis examined the effect of *CTRP6* overexpression on the AMPK/SIRT1/PGC‐1α signalling pathway‐related proteins including p‐AMPK, AMPK, SIRT1 and PGC‐1α protein levels in H9c2 cardiomyocytes in the presence of ISO (*n* = 3). (e) Flow cytometry analysed the effect of *CTRP6* overexpression on cell apoptosis in H9c2 cardiomyocytes in the presence of ISO (*n* = 3). (f) Phalloidin staining to visualize F‐actin expression in H9c2 cardiomyocytes in the control, ISO, ISO+Oe‐NC and ISO+Oe‐*CTRP6* groups (*n* = 3) using fluorescent phalloidin (green). Scale bar = 100 µm. (g) RT‐qPCR analysed the effect of *CTRP6* overexpression on levels of hypertrophic markers including *ANP*, *BNP* and *β‐MHC* in H9c2 cardiomyocytes in the presence of ISO (*n* = 3).

Mitochondria serve as downstream targets of AMPK (Cai et al., 2020), with the AMPK/SIRT1/PGC‐1α signalling pathway playing a pivotal role in mitochondrial function, OS, and apoptosis regulation (Gao et al., 2020). To investigate whether *CTRP6* regulates HF progression via this pathway, we examined major pathway proteins in heart tissues. Our results revealed that ISO induction significantly reduced the levels of phosphorylated AMPK (p‐AMPK), SIRT1 and PGC‐1α, indicative of pathway suppression. Conversely, *CTRP6* overexpression restored the expression of these proteins (Figure 4a), suggesting that *CTRP6* activates the AMPK/SIRT1/PGC‐1α signalling pathway to protect mitochondrial function. Given that cardiomyocyte apoptosis contributes to cardiac dysfunction, we investigated the in vitro effects of *CTRP6* on cardiomyocytes by transfecting cells with an oe‐*CTRP6* overexpression vector and treating them with ISO solution. RT‐qPCR confirmed that after transfection with an oe‐*CTRP6* overexpression vector, *CTRP6* expression was elevated in H9c2 cardiomyocytes in the presence of ISO (Figure 4b). CCK‐8 assay results demonstrated that ISO treatment suppressed H9c2 cell activity, whereas *CTRP6* overexpression enhanced cardiomyocyte activity (Figure 4c). Additionally, Western blot analysis confirmed that *CTRP6* overexpression activated the AMPK/SIRT1/PGC‐1α pathway, which was repressed by ISO (Figure 4d). Further experiments revealed that ISO‐induced apoptosis was attenuated by *CTRP6* overexpression, but this effect was reversed by treatment with Compound C (CC), an AMPK inhibitor (Figure 4e). Moreover, phalloidin staining demonstrated that *CTRP6* overexpression significantly reduced the increased cell surface area induced by ISO, an effect reversed by CC treatment (Figure 4f). RT‐qPCR analysis corroborated these findings, showing that ISO upregulated hypertrophic markers *ANP*, *BNP* and *β‐MHC* expression, which was inhibited by *CTRP6* overexpression, yet reversed by CC treatment (Figure 4g). In conclusion, *CTRP6* attenuates ISO‐induced hypertrophy and apoptosis in H9c2 cardiomyocytes through the AMPK/SIRT1/PGC‐1α pathway.

### 3.5 *CTRP6* attenuates OS, inflammatory cytokines level and mitochondrial dysfunction in ISO‐induced H9c2 cardiomyocytes

**FIGURE 5:**
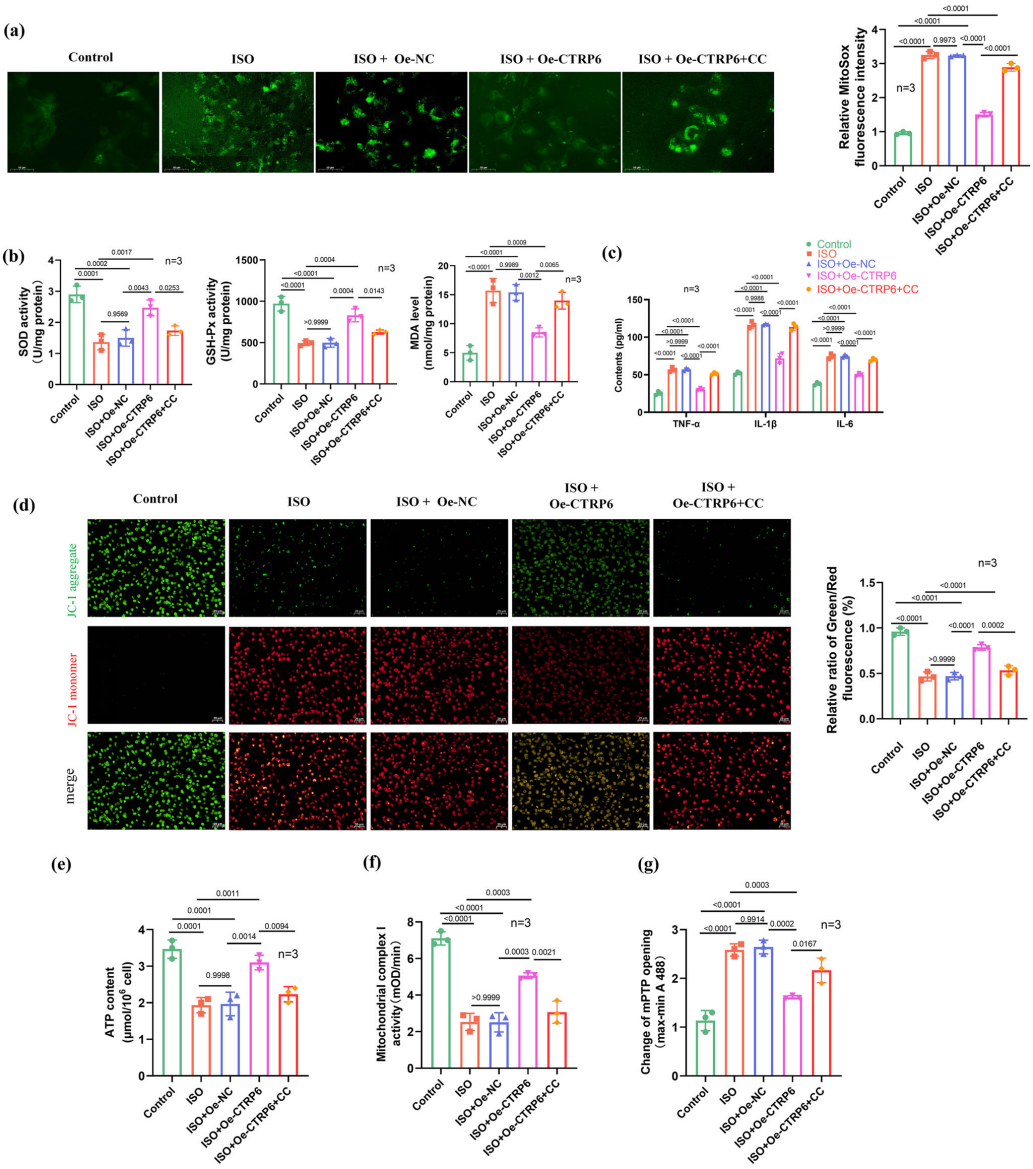
*CTRP6* attenuates OS, inflammation and mitochondrial dysfunction in ISO‐induced H9c2 cardiomyocytes via activating the AMPK/SIRT1/PGC‐1α pathway. (a) DCFH‐DA fluorescent probe was used to assess ROS production in cells in the control, ISO, ISO+Oe‐NC, ISO+Oe‐*CTRP6* and ISO+Oe‐*CTRP6*+CC groups (*n* = 3). CC is an AMPK inhibitor. Scale bar = 100 µm. (b) Determination of MDA, GSH‐Px and SOD concentrations in the control, ISO, ISO+Oe‐NC, ISO+Oe‐*CTRP6* and ISO+Oe‐*CTRP6*+CC groups (*n* = 3). (c) ELISA‐examined levels of inflammatory factors including *TNF‐α*, *IL‐1β* and *IL‐6* in the control, ISO, ISO+Oe‐NC, ISO+Oe‐*CTRP6* and ISO+Oe‐*CTRP6*+CC groups (*n* = 3). (d) Assessment of MMP loss using the JC‐1 assay in the control, ISO, ISO+Oe‐NC, ISO+Oe‐*CTRP6* and ISO+Oe‐*CTRP6*+CC groups (*n* = 3). Scale bar = 100 μm. (e) Measurement of ATP content in the control, ISO, ISO+Oe‐NC, ISO+Oe‐*CTRP6* and ISO+Oe‐*CTRP6*+CC groups (*n* = 3). (f) Evaluation of mitochondrial complex I activity in the control, ISO, ISO+Oe‐NC, ISO+Oe‐*CTRP6* and ISO+Oe‐*CTRP6*+CC groups (*n* = 3). (g) Testing of mPTP opening in the control, ISO, ISO+Oe‐NC, ISO+Oe‐*CTRP6* and ISO+Oe‐*CTRP6*+CC groups (*n* = 3).

To further elucidate the effects of *CTRP6* on OS, inflammatory cytokine levels and mitochondrial function in cardiomyocytes, we conducted a series of experiments. We utilized the DCFH‐DA fluorescent probe to assess intracellular ROS levels. Our results demonstrated that ISO stimulation significantly increased ROS levels, which were effectively attenuated by CTRP6 overexpression. However, this inhibitory effect of *CTRP6* on ROS levels was counteracted by treatment with CC, an AMPK inhibitor (Figure 5a). Furthermore, we investigated the levels of SOD and GSH‐Px, as well as MDA. ISO treatment led to a decrease in SOD and GSH‐Px concentrations, accompanied by an increase in MDA content. Remarkably, *CTRP6* overexpression reversed these effects, restoring SOD and GSH‐Px levels while reducing MDA content. However, CC treatment effectively reversed the effects of *CTRP6* overexpression on SOD, GSH‐Px and MDA levels (Figure 5b). At the same time, we observed that ISO treatment led to an increase in TNF‐α, IL‐1β and IL‐6 contents, while *CTRP6* overexpression reversed these effects, reducing *TNF‐α*, *IL‐1β* and *IL‐6* contents. However, CC treatment effectively reversed the effects of *CTRP6* overexpression on *TNF‐α*, *IL‐1β* and *IL‐6* contents (Figure 5c). We also assessed mitochondrial membrane potential (MMP) using JC‐1 staining to investigate mitochondrial health. ISO stimulation resulted in MMP loss, whereas *CTRP6* overexpression rescued ISO‐induced MMP loss. However, CC treatment reversed the rescuing effect of *CTRP6* overexpression (Figure 5d). In addition, we examined ATP content and complex I activity in H9c2 cardiomyocytes. ISO treatment led to a decrease in ATP content and complex I activity, which were restored by *CTRP6* overexpression. However, CC treatment suppressed the effects of *CTRP6* overexpression on ATP content and complex I activity (Figure 5e,f). Finally, we evaluated the opening of the mPTP. ISO‐induced mPTP opening was attenuated by *CTRP6* overexpression, whereas CC treatment neutralized the effect of *CTRP6* overexpression on mPTP opening (Figure 5g). These findings collectively indicate that *CTRP6* attenuates oxidative stress, inflammation and mitochondrial dysfunction by activating the AMPK/SIRT1/PGC‐1α pathway in ISO‐induced H9c2 cardiomyocytes.

## 4 DISCUSSION

HF presents a significant healthcare challenge, affecting an estimated 64.3 million individuals globally, with a notable increase observed over recent decades (GBD 2016 Disease & Injury Incidence & Prevalence Collaborators, 2017). Therefore, developing new therapeutic strategies for HF is paramount. In this study, we used ISO‐induced HFrEF mouse models, as ISO mimics pathological changes akin to human HF (Ren et al., 2019). Echocardiography, a reliable non‐invasive diagnostic tool, indicated impaired left ventricular systolic function in the HFrEF mice, confirming successful model induction (Liu et al., 2022).

*CTRP6*, a member of the *CTRP* family associated with adiponectin, has shown promise in modulating cardiovascular diseases (Hu et al., 2022; Xie et al., 2021; Zhang et al., 2022). Our investigation revealed low *CTRP6* expression in HF patients and demonstrated that its upregulation in HFrEF mice ameliorated cardiac dysfunction and pathological changes, suggesting a cardioprotective role (Liu et al., 2023; Niemann et al., 2020; Peng et al., 2020). Studies have revealed that during HF advancement, cardiomyocyte death progresses slowly yet steadily, resulting in ongoing loss (Narula et al., 1996). Our experimental findings corroborated this, showing that ISO induction heightened apoptosis in both cardiac tissues and H9c2 cardiomyocytes. Remarkably, *CTRP6* overexpression counteracted this effect, effectively preventing apoptosis. Hence, we concluded that *CTRP6* significantly ameliorated cardiac injury and exerted cardioprotective effects in HFrEF mice.

OS plays a pivotal role in HF development, inducing apoptosis, necrosis, inflammation and fibrosis in cardiomyocytes (van der Pol et al., 2019). It arises from disruptions in the antioxidant defence system due to excess ROS production or inadequate antioxidant activity, exacerbating cardiac insufficiency and cardiomyocyte damage (Puente et al., 2014). In our study, ISO induction triggered ROS accumulation in the mouse heart, leading to lipid peroxidation and a significant increase in myocardial MDA content. Concurrently, the activity of common antioxidant enzymes, SOD and GSH, decreased as OS intensified (Apel & Hirt, 2004; Marrocco et al., 2017; Senoner & Dichtl, 2019). Notably, *CTRP6* overexpression mitigated ROS and MDA levels while enhancing SOD and GSH levels in myocardial tissues of ISO‐induced model mice, a finding consistent with our observations in an HFrEF cell model. Thus, our data suggest that *CTRP6* alleviates HF progression by suppressing OS. Moreover, previous studies have corroborated our findings, demonstrating that *CTRP6* attenuates cerebral ischaemia–reperfusion injury and renal injury by reducing ROS and MDA levels, further supporting the therapeutic potential of *CTRP6* in combating OS‐related pathologies (Li et al., 2020; Xiang et al., 2020; Zhao et al., 2024). Moreover, inflammation is widely perceived as a dominating risk factor for HF (Adamo et al., 2020). In the current study, overexpressed *CTRP6* decreased levels of pro‐inflammatory cytokines (*TNF‐α*, *IL‐1β* and *IL‐6*) in ISO‐induced H9c2 cardiomyocytes, indicating mitigation of inflammation. Consistent with this, it has been reported that *CTRP6* attenuates cerebral ischaemia–reperfusion‑induced inflammation, oxidative stress and apoptosis (Li et al., 2020). Mitochondria, dynamic organelles integral to cellular processes, serve as primary sources of superoxide production, chiefly through the electron transport chain's complex I and complex III (Chen et al., 2003; Hirst et al., 2008). Impaired mitochondrial function is a hallmark of metabolic, cardiovascular and neurological disorders (Amorim et al., 2022; Chistiakov et al., 2018; Whitley et al., 2019). This dysfunction manifests through the persistent opening of the mitochondrial mPTP, release of cytochrome *c*, overproduction of mitochondrial ROS, diminished ATP synthesis and compromised mitochondrial membrane potential, culminating in apoptosis (Brand & Nicholls, 2011). Notably, modulation of cardiomyocyte differentiation and metabolic pathways via mPTP opening underscores its pivotal role in cardiac function (Hom et al., 2011; Hrstka et al., 2017). Recent studies have highlighted diverse mechanisms influencing mitochondrial function in cardiac health. *Circ‐Samd4* and *LARP7*, for example, exhibit protective effects against HF by modulating mitochondrial processes (Yu et al., 2021; Zheng et al., 2022). Mitochondrial fatty acid β‐oxidation (FAO) is the major pathway for the degradation of fatty acids and is essential for maintaining energy homeostasis in the human body (Houten et al., 2016). It has been reported that *CTRP6* plays a key role in kidney fibrosis by promoting FAO (Xie et al., 2021), highlighting the critical role of *CTRP6* in maintaining mitochondrial activity. Importantly, our investigation revealed that *CTRP6* overexpression significantly reduces mitochondrial ROS production, preserves membrane potential and inhibits mPTP opening, while enhancing ATP production and activity of mitochondrial complexes I and III. These findings underscore the potential of *CTRP6* in maintaining mitochondrial homeostasis, presenting a promising avenue for HF management.

PGC‐1α, a key player in cellular energy regulation and mitochondrial remodelling, combats OS (Wu et al., 1999). AMPK, an energy sensor, maintains cellular energetic balance and activates mitochondrial biogenesis through PGC‐1α (Hardie et al., 2012). SIRT1, part of the sirtuin family, regulates metabolism and mitochondrial biogenesis (Cantó & Auwerx, 2009). Activation of PGC‐1α requires AMPK phosphorylation and SIRT1 deacetylation, with the AMPK/SIRT1/PGC‐1α pathway implicated in various human diseases (Lee et al., 2021; Valero, 2014; Zhang et al., 2021). For instance, *MC1R* regulates brain injury by modulating mitochondrial metabolism through this pathway (Xu et al., 2021), while acacetin alleviates cardiac hypertrophy via AMPK/SIRT1/PGC‐1α (Cui et al., 2022). Linggui Zhugan suppresses mitochondrial damage in HF rats by activating the same pathway (Yu et al., 2023). Additionally, *CTRP6* has been shown to mitigate *TNF‐α*‐induced salivary gland vesicle cell apoptosis by activating AMPK/SIRT1 signalling (Qu et al., 2021). Moreover, *CTRP6* activates AMPK/SIRT1/PGC‐1α, as demonstrated in our study. In rescue assays, the AMPK/SIRT1 inhibitor CC reversed the suppressive effect of *CTRP6* upregulation on cell apoptosis, hypertrophy, OS and mitochondrial dysfunction in ISO‐treated H9c2 cardiomyocytes.

There are some limitations to our study. First, we used plasma samples from patients without collecting and analysing myocardial tissues, which would provide more convincing results. Second, the sample size is limited, which may affect the accuracy of our findings. Additionally, the correlation between *CTRP6* expression and the survival of HF patients was not investigated. Therefore, future studies should adopt a larger sample size and conduct more in‐depth research to address these limitations.

In summary, our findings highlight that *CTRP6* enhances mitochondrial homeostasis and reduces cardiomyocyte apoptosis, thus alleviating HF progression via the AMPK/SIRT1/PGC‐1α signalling pathway. These insights may unveil novel targets for HF treatment.

## AUTHOR CONTRIBUTIONS

Xianhe Lin contributed to the conception and design of the research. Ningjun Zhu performed the experiments. Mengli Li and Zhen Wang analysed the data and interpreted the results of the experiments. Tingting Fan drafted a revised manuscript. All authors have read and approved the final version of this manuscript and agree to be accountable for all aspects of the work in ensuring that questions related to the accuracy or integrity of any part of the work are appropriately investigated and resolved. All persons designated as authors qualify for authorship, and all those who qualify for authorship are listed.

## CONFLICT OF INTEREST

The authors declare they have no conflicts of interest.

## Data Availability

The data supporting the findings of this study are available from the corresponding author upon reasonable request.

## References

[eph13658-bib-0001] Adamo, L. , Rocha‐Resende, C. , Prabhu, S. D. , & Mann, D. L. (2020). Reappraising the role of inflammation in heart failure. Nature Reviews Cardiology, 17(5), 269–285. 31969688 10.1038/s41569-019-0315-x

[eph13658-bib-0002] Amorim, J. A. , Coppotelli, G. , Rolo, A. P. , Palmeira, C. M. , Ross, J. M. , & Sinclair, D. A. (2022). Mitochondrial and metabolic dysfunction in ageing and age‐related diseases. Nature Reviews Endocrinology, 18(4), 243–258. 10.1038/s41574-021-00626-7 PMC9059418 35145250

[eph13658-bib-0003] Apel, K. , & Hirt, H. (2004). Reactive oxygen species: Metabolism, oxidative stress, and signal transduction. Annual Review of Plant Biology, 55(1), 373–399. 10.1146/annurev.arplant.55.031903.141701 15377225

[eph13658-bib-0004] Atici, A. E. , Crother, T. R. , & Noval Rivas, M. (2023). Mitochondrial quality control in health and cardiovascular diseases. Frontiers in Cell and Developmental Biology, 11, 1290046. 38020895 10.3389/fcell.2023.1290046 PMC10657886

[eph13658-bib-0005] Bacmeister, L. , Schwarzl, M. , Warnke, S. , Stoffers, B. , Blankenberg, S. , Westermann, D. , & Lindner, D. (2019). Inflammation and fibrosis in murine models of heart failure. Basic Research in Cardiology, 114(3), 19. 30887214 10.1007/s00395-019-0722-5

[eph13658-bib-0006] Brand, M. D. , & Nicholls, D. G. (2011). Assessing mitochondrial dysfunction in cells. Biochemical Journal, 435(2), 297–312. 21726199 10.1042/BJ20110162 PMC3076726

[eph13658-bib-0007] Cai, Z. , Li, C. F. , Han, F. , Liu, C. , Zhang, A. , Hsu, C. C. , Peng, D. , Zhang, X. , Jin, G. , Rezaeian, A. H. , Wang, G. , Zhang, W. , Pan, B. S. , Wang, C. Y. , Wang, Y. H. , Wu, S. Y. , Yang, S. C. , Hsu, F. C. , D'Agostino, R. B., Jr , … Lin, H. K. (2020). Phosphorylation of PDHA by AMPK drives TCA cycle to promote cancer metastasis. Molecular Cell, 80(2), 263–278.e7. 33022274 10.1016/j.molcel.2020.09.018 PMC7534735

[eph13658-bib-0008] Cantó, C. , & Auwerx, J. (2009). PGC‐1alpha, SIRT1 and AMPK, an energy sensing network that controls energy expenditure. Current Opinion in Lipidology, 20(2), 98–105. 19276888 10.1097/MOL.0b013e328328d0a4 PMC3627054

[eph13658-bib-0009] Chen, Q. , Vazquez, E. J. , Moghaddas, S. , Hoppel, C. L. , & Lesnefsky, E. J. (2003). Production of reactive oxygen species by mitochondria: Central role of complex III. Journal of Biological Chemistry, 278(38), 36027–36031. 12840017 10.1074/jbc.M304854200

[eph13658-bib-0010] Chistiakov, D. A. , Shkurat, T. P. , Melnichenko, A. A. , Grechko, A. V. , & Orekhov, A. N. (2018). The role of mitochondrial dysfunction in cardiovascular disease: A brief review. Annals of Medicine, 50(2), 121–127. 29237304 10.1080/07853890.2017.1417631

[eph13658-bib-0011] Cui, Y. K. , Hong, Y. X. , Wu, W. Y. , Han, W. M. , Wu, Y. , Wu, C. , Li, G. R. , & Wang, Y. (2022). Acacetin ameliorates cardiac hypertrophy by activating Sirt1/AMPK/PGC‐1α pathway. European Journal of Pharmacology, 920, 174858. 35219729 10.1016/j.ejphar.2022.174858

[eph13658-bib-0012] Di Cesare, M. , Perel, P. , Taylor, S. , Kabudula, C. , Bixby, H. , Gaziano, T. A. , McGhie, D. V. , Mwangi, J. , Pervan, B. , Narula, J. , Pineiro, D. , & Pinto, F. J. (2024). The heart of the world. Global Heart, 19(1), 11. 38273998 10.5334/gh.1288 PMC10809869

[eph13658-bib-0013] Gao, J. , Qian, T. , & Wang, W. (2020). CTRP3 activates the AMPK/SIRT1‐PGC‐1α pathway to protect mitochondrial biogenesis and functions in cerebral ischemic stroke. Neurochemical Research, 45(12), 3045–3058. 33098065 10.1007/s11064-020-03152-6

[eph13658-bib-0014] GBD 2016 Disease and Injury Incidence and Prevalence Collaborators . (2017). Global, regional, and national incidence, prevalence, and years lived with disability for 328 diseases and injuries for 195 countries, 1990–2016: A systematic analysis for the Global Burden of Disease Study 2016. The Lancet, 390(10100), 1211–1259. 10.1016/S0140-6736(17)32154-2 PMC5605509 28919117

[eph13658-bib-0015] Grundy, D. (2015). Principles and standards for reporting animal experiments in *The Journal of Physiology* and *Experimental Physiology* . Experimental Physiology, 100, 755–758. 26076765 10.1113/EP085299

[eph13658-bib-0016] Hardie, D. G. , Ross, F. A. , & Hawley, S. A. (2012). AMPK: A nutrient and energy sensor that maintains energy homeostasis. Nature Reviews Molecular Cell Biology, 13(4), 251–262. 22436748 10.1038/nrm3311 PMC5726489

[eph13658-bib-0017] Hirst, J. , King, M. S. , & Pryde, K. R. (2008). The production of reactive oxygen species by complex I. Biochemical Society Transactions, 36(Pt 5), 976–980. 18793173 10.1042/BST0360976

[eph13658-bib-0018] Hom, J. R. , Quintanilla, R. A. , Hoffman, D. L. , de Mesy Bentley, K. L. , Molkentin, J. D. , Sheu, S. S. , & Porter, G. A., Jr. (2011). The permeability transition pore controls cardiac mitochondrial maturation and myocyte differentiation. Developmental Cell, 21(3), 469–478. 21920313 10.1016/j.devcel.2011.08.008 PMC3175092

[eph13658-bib-0019] Houten, S. M. , Violante, S. , Ventura, F. V. , & Wanders, R. J. (2016). The biochemistry and physiology of mitochondrial fatty acid β‐oxidation and its genetic disorders. Annual Review of Physiology, 78(1), 23–44. 10.1146/annurev-physiol-021115-105045 26474213

[eph13658-bib-0020] Hrstka, S. C. , Li, X. , & Nelson, T. J. (2017). NOTCH1‐dependent nitric oxide signaling deficiency in hypoplastic left heart syndrome revealed through patient‐specific phenotypes detected in bioengineered cardiogenesis. Stem Cells, 35(4), 1106–1119. 28142228 10.1002/stem.2582

[eph13658-bib-0021] Hu, B. , Qian, X. , Qian, P. , Xu, G. , Jin, X. , Chen, D. , Xu, L. , Tang, J. , Wu, W. , Li, W. , & Zhang, J. (2022). Advances in the functions of CTRP6 in the development and progression of the malignancy. Frontiers in Genetics, 13, 985077. 36313428 10.3389/fgene.2022.985077 PMC9596804

[eph13658-bib-0022] Jin, W. , Zhang, Y. , Xue, Y. , Han, X. , Zhang, X. , Ma, Z. , Sun, S. , Chu, X. , Cheng, J. , Guan, S. , Li, Z. , & Chu, L. (2020). Crocin attenuates isoprenaline‐induced myocardial fibrosis by targeting TLR4/NF‐κB signaling: Connecting oxidative stress, inflammation, and apoptosis. Naunyn‐Schmiedebergs Archives of Pharmacology, 393(1), 13–23. 31392383 10.1007/s00210-019-01704-4

[eph13658-bib-0023] Lee, G. H. , Peng, C. , Lee, H. Y. , Park, S. A. , Hoang, T. H. , Kim, J. H. , Sa, S. , Kim, G. E. , Han, J. S. , & Chae, H. J. (2021). D‐allulose ameliorates adiposity through the AMPK‐SIRT1‐PGC‐1α pathway in HFD‐induced SD rats. Food & Nutrition Research, 65. 10.29219/fnr.v65.7803 PMC8829832 35221861

[eph13658-bib-0024] Lee, J. H. , Kim, D. H. , Kim, M. , Jung, K. H. , & Lee, K. H. (2022). Mitochondrial ROS‐mediated metabolic and cytotoxic effects of isoproterenol on cardiomyocytes are p53‐dependent and reversed by curcumin. Molecules, 27(4), 1346. 35209134 10.3390/molecules27041346 PMC8877017

[eph13658-bib-0025] Lei, H. , Wu, D. , Wang, J. Y. , Li, L. , Zhang, C. L. , Feng, H. , Fu, F. Y. , & Wu, L. L. (2015). C1q/tumor necrosis factor‐related protein‐6 attenuates post‐infarct cardiac fibrosis by targeting RhoA/MRTF‐A pathway and inhibiting myofibroblast differentiation. Basic Research in Cardiology, 110(4), 35. 25962701 10.1007/s00395-015-0492-7

[eph13658-bib-0026] Li, Y. , Chen, B. , Yang, X. , Zhang, C. , Jiao, Y. , Li, P. , Liu, Y. , Li, Z. , Qiao, B. , Bond Lau, W. , Ma, X. L. , & Du, J. (2019). S100a8/a9 signaling causes mitochondrial dysfunction and cardiomyocyte death in response to ischemic/reperfusion injury. Circulation, 140(9), 751–764. 31220942 10.1161/CIRCULATIONAHA.118.039262

[eph13658-bib-0027] Li, Y. , Sun, J. , Gu, L. , & Gao, X. (2020). Protective effect of CTRP6 on cerebral ischemia/reperfusion injury by attenuating inflammation, oxidative stress and apoptosis in PC12 cells. Molecular Medicine Reports, 22(1), 344–352. 32377750 10.3892/mmr.2020.11108 PMC7248524

[eph13658-bib-0028] Liang, S. , Han, J. , Cheng, W. , & Chen, X. (2023). C1q/tumor necrosis factor‐related protein‐6 exerts protective effects on myocardial ischemia‐reperfusion injury through the modulation of the Akt‐GSK‐3β‐Nrf2 signaling cascade. International Immunopharmacology, 115, 109678. 36634414 10.1016/j.intimp.2023.109678

[eph13658-bib-0029] Lindstrom, M. , DeCleene, N. , Dorsey, H. , Fuster, V. , Johnson, C. O. , LeGrand, K. E. , Mensah, G. A. , Razo, C. , Stark, B. , Varieur Turco, J. , & Roth, G. A. (2022). Global burden of cardiovascular diseases and risks collaboration, 1990–2021. Journal of the American College of Cardiology, 80(25), 2372–2425. 36517116 10.1016/j.jacc.2022.11.001

[eph13658-bib-0030] Liu, F. , Di, Y. , Ma, W. , Kang, X. , Li, X. , & Ji, Z. (2022). HDAC9 exacerbates myocardial infarction via inactivating Nrf2 pathways. Journal of Pharmacy and Pharmacology, 74(4), 565–572. 33963859 10.1093/jpp/rgab065

[eph13658-bib-0031] Liu, Y. , Wu, P. , Xu, X. , Shen, T. , Wang, X. , Liu, Y. , Yuan, C. , Wang, T. , Zhou, L. , & Liu, A. (2023). C1q/TNF‐related protein 3 alleviates heart failure via attenuation of oxidative stress in myocardial infarction rats. Peptides, 163, 170980. 36842629 10.1016/j.peptides.2023.170980

[eph13658-bib-0032] MacDonald, B. J. , Virani, S. A. , Zieroth, S. , & Turgeon, R. (2023). Heart failure management in 2023: A pharmacotherapy‐ and lifestyle‐focused comparison of current international guidelines. CJC Open, 5(8), 629–640. 37720183 10.1016/j.cjco.2023.05.008 PMC10502425

[eph13658-bib-0033] Marrocco, I. , Altieri, F. , & Peluso, I. (2017). Measurement and clinical significance of biomarkers of oxidative stress in humans. Oxidative Medicine and Cellular Longevity, 2017(1), 6501046. 28698768 10.1155/2017/6501046 PMC5494111

[eph13658-bib-0034] Narula, J. , Haider, N. , Virmani, R. , DiSalvo, T. G. , Kolodgie, F. D. , Hajjar, R. J. , Schmidt, U. , Semigran, M. J. , Dec, G. W. , & Khaw, B. A. (1996). Apoptosis in myocytes in end‐stage heart failure. New England Journal of Medicine, 335(16), 1182–1189. 8815940 10.1056/NEJM199610173351603

[eph13658-bib-0035] Niemann, B. , Li, L. , Siegler, D. , Siegler, B. H. , Knapp, F. , Hanna, J. , Aslam, M. , Kracht, M. , Schulz, R. , & Rohrbach, S. (2020). CTRP9 mediates protective effects in cardiomyocytes via AMPK‐ and adiponectin receptor‐mediated induction of anti‐oxidant response. Cells, 9(5), 1229. 32429302 10.3390/cells9051229 PMC7291146

[eph13658-bib-0036] Pang, Y. , Huang, M. , Lu, J. , Peng, Z. , Tang, M. , Huang, P. , Zhai, Y. , & Lu, J. (2023). Global trends in research on oxidative stress related to heart failure from 2012 to 2021: A bibliometric analysis and suggestion to researchers. Annals of Translational Medicine, 11(2), 54. 36819531 10.21037/atm-22-6573 PMC9929796

[eph13658-bib-0037] Peng, M. , Liu, Y. , Zhang, X. Q. , Xu, Y. W. , Zhao, Y. T. , & Yang, H. B. (2020). CTRP5‐overexpression attenuated ischemia‐reperfusion associated heart injuries and improved infarction induced heart failure. Frontiers in Pharmacology, 11, 603322. 33414720 10.3389/fphar.2020.603322 PMC7783420

[eph13658-bib-0038] Puente, B. N. , Kimura, W. , Muralidhar, S. A. , Moon, J. , Amatruda, J. F. , Phelps, K. L. , Grinsfelder, D. , Rothermel, B. A. , Chen, R. , Garcia, J. A. , Santos, C. X. , Thet, S. , Mori, E. , Kinter, M. T. , Rindler, P. M. , Zacchigna, S. , Mukherjee, S. , Chen, D. J. , Mahmoud, A. I. , … Sadek, H. A. (2014). The oxygen‐rich postnatal environment induces cardiomyocyte cell‐cycle arrest through DNA damage response. Cell, 157(3), 565–579. 24766806 10.1016/j.cell.2014.03.032 PMC4104514

[eph13658-bib-0039] Qu, L. H. , Hong, X. , Zhang, Y. , Cong, X. , Xiang, R. L. , Mei, M. , Su, J. Z. , Wu, L. L. , & Yu, G. Y. (2021). C1q/tumor necrosis factor‐related protein‐6 attenuates TNF‐α‐induced apoptosis in salivary acinar cells via AMPK/SIRT1‐modulated miR‐34a‐5p expression. Journal of Cellular Physiology, 236(8), 5785–5800. 33400820 10.1002/jcp.30262

[eph13658-bib-0040] Ren, S. , Chang, S. , Tran, A. , Mandelli, A. , Wang, Y. , & Wang, J. J. (2019). Implantation of an isoproterenol mini‐pump to induce heart failure in mice. Journal of Visualized Experiments, (152), 10.3791/59646 PMC7374011 31633680

[eph13658-bib-0041] Scarà, A. , Palamà, Z. , Robles, A. G. , Dei, L. L. , Borrelli, A. , Zanin, F. , Pignalosa, L. , Romano, S. , & Sciarra, L. (2024). Non‐pharmacological treatment of heart failure‐from physical activity to electrical therapies: A literature review. Journal of Cardiovascular Development and Disease, 11(4), 122. 38667740 10.3390/jcdd11040122 PMC11050051

[eph13658-bib-0042] Schwinger, R. H. G. (2021). Pathophysiology of heart failure. Cardiovascular Diagnosis and Therapy, 11(1), 263–276. 33708498 10.21037/cdt-20-302 PMC7944197

[eph13658-bib-0043] Senoner, T. , & Dichtl, W. (2019). Oxidative stress in cardiovascular diseases: Still a therapeutic target? Nutrients, 11(9), 2090. 31487802 10.3390/nu11092090 PMC6769522

[eph13658-bib-0044] Snipelisky, D. , Chaudhry, S. P. , & Stewart, G. C. (2019). The many faces of heart failure. Cardiac Electrophysiology Clinics, 11(1), 11–20. 30717842 10.1016/j.ccep.2018.11.001

[eph13658-bib-0045] Tomasoni, D. , Adamo, M. , Lombardi, C. M. , & Metra, M. (2019). Highlights in heart failure. ESC Heart Failure, 6(6), 1105–1127. 31997538 10.1002/ehf2.12555 PMC6989277

[eph13658-bib-0046] Tsutsui, H. (2022). Recent advances in the pharmacological therapy of chronic heart failure: Evidence and guidelines. Pharmacology & Therapeutics, 238, 108185. 35413307 10.1016/j.pharmthera.2022.108185

[eph13658-bib-0047] Tsutsui, H. , Kinugawa, S. , & Matsushima, S. (2011). Oxidative stress and heart failure. American Journal of Physiology‐Heart and Circulatory Physiology, 301(6), H2181–H2190. 21949114 10.1152/ajpheart.00554.2011

[eph13658-bib-0048] Valero, T. (2014). Mitochondrial biogenesis: Pharmacological approaches. Current Pharmaceutical Design, 20(35), 5507–5509. 24606795 10.2174/138161282035140911142118

[eph13658-bib-0049] van der Pol, A. , van Gilst, W. H. , Voors, A. A. , & van der Meer, P. (2019). Treating oxidative stress in heart failure: Past, present and future. European Journal of Heart Failure, 21(4), 425–435. 30338885 10.1002/ejhf.1320 PMC6607515

[eph13658-bib-0050] Wang, R. , Luo, X. , Li, S. , Wen, X. , Zhang, X. , Zhou, Y. , & Xie, W. (2023). A bibliometric analysis of cardiomyocyte apoptosis from 2014 to 2023: A review. Medicine, 102(47), e35958. 38013295 10.1097/MD.0000000000035958 PMC10681623

[eph13658-bib-0051] Whitley, B. N. , Engelhart, E. A. , & Hoppins, S. (2019). Mitochondrial dynamics and their potential as a therapeutic target. Mitochondrion, 49, 269–283. 31228566 10.1016/j.mito.2019.06.002 PMC6885535

[eph13658-bib-0052] Wong, G. W. , Krawczyk, S. A. , Kitidis‐Mitrokostas, C. , Revett, T. , Gimeno, R. , & Lodish, H. F. (2008). Molecular, biochemical and functional characterizations of C1q/TNF family members: Adipose‐tissue‐selective expression patterns, regulation by PPAR‐gamma agonist, cysteine‐mediated oligomerizations, combinatorial associations and metabolic functions. Biochemical Journal, 416(2), 161–177. 18783346 10.1042/BJ20081240 PMC3936483

[eph13658-bib-0053] Wu, Z. , Puigserver, P. , Andersson, U. , Zhang, C. , Adelmant, G. , Mootha, V. , Troy, A. , Cinti, S. , Lowell, B. , Scarpulla, R. C. , & Spiegelman, B. M. (1999). Mechanisms controlling mitochondrial biogenesis and respiration through the thermogenic coactivator PGC‐1. Cell, 98(1), 115–124. 10412986 10.1016/S0092-8674(00)80611-X

[eph13658-bib-0054] Xiang, H. , Xue, W. , Li, Y. , Zheng, J. , Ding, C. , Dou, M. , & Wu, X. (2020). C1q/TNF‐related protein 6 (CTRP6) attenuates renal ischaemia‐reperfusion injury through the activation of PI3K/Akt signalling pathway. Clinical and Experimental Pharmacology & Physiology, 47(6), 1030–1040. 32027040 10.1111/1440-1681.13274

[eph13658-bib-0055] Xie, Y. H. , Xiao, Y. , Huang, Q. , Hu, X. F. , Gong, Z. C. , & Du, J. (2021). Role of the CTRP6/AMPK pathway in kidney fibrosis through the promotion of fatty acid oxidation. European Journal of Pharmacology, 892, 173755. 33245899 10.1016/j.ejphar.2020.173755

[eph13658-bib-0056] Xu, C. , Sarver, D. C. , Lei, X. , Sahagun, A. , Zhong, J. , Na, C. H. , Rudich, A. , & Wong, G. W. (2024). CTRP6 promotes the macrophage inflammatory response, and its deficiency attenuates LPS‐induced inflammation. Journal of Biological Chemistry, 300(1), 105566. 38103643 10.1016/j.jbc.2023.105566 PMC10789631

[eph13658-bib-0057] Xu, W. , Yan, J. , Ocak, U. , Lenahan, C. , Shao, A. , Tang, J. , Zhang, J. , & Zhang, J. H. (2021). Melanocortin 1 receptor attenuates early brain injury following subarachnoid hemorrhage by controlling mitochondrial metabolism via AMPK/SIRT1/PGC‐1α pathway in rats. Theranostics, 11(2), 522–539. 33391490 10.7150/thno.49426 PMC7738864

[eph13658-bib-0058] Ye, Y. , Jia, X. , Bajaj, M. , & Birnbaum, Y. (2018). Dapagliflozin attenuates Na(+)/H(+) exchanger‐1 in cardiofibroblasts via AMPK activation. Cardiovascular Drugs and Therapy, 32(6), 553–558. 30367338 10.1007/s10557-018-6837-3

[eph13658-bib-0059] Yu, H. , Zhang, F. , Yan, P. , Zhang, S. , Lou, Y. , Geng, Z. , Li, Z. , Zhang, Y. , Xu, Y. , Lu, Y. , Chen, C. , Wang, D. , Zhu, W. , Hu, X. , Wang, J. , Zhuang, T. , Zhang, Y. , Wu, G. , Liu, J. , … Zhang, B. (2021). LARP7 protects against heart failure by enhancing mitochondrial biogenesis. Circulation, 143(20), 2007–2022. 33663221 10.1161/CIRCULATIONAHA.120.050812

[eph13658-bib-0060] Yu, S. , Qian, H. , Tian, D. , Yang, M. , Li, D. , Xu, H. , Chen, J. , Yang, J. , Hao, X. , Liu, Z. , Zhong, J. , Yang, H. , Chen, X. , Min, X. , & Chen, J. (2023). Linggui Zhugan Decoction activates the SIRT1‐AMPK‐PGC1α signaling pathway to improve mitochondrial and oxidative damage in rats with chronic heart failure caused by myocardial infarction. Frontiers in Pharmacology, 14, 1074837. 37089931 10.3389/fphar.2023.1074837 PMC10113531

[eph13658-bib-0061] Zhang, A. , Kong, M. , Zhang, X. , & Pei, Z. (2022). Mechanism of action of CTRP6 in the regulation of tumorigenesis in the digestive system. Oncology Letters, 24(5), 391. 36276484 10.3892/ol.2022.13511 PMC9533366

[eph13658-bib-0062] Zhang, C. L. , Wu, L. L. , & Li, L. (2017). [Research progress of complement‐C1q/tumor necrosis factor‐related protein 3]. Sheng Li Xue Bao [Acta Physiologica Sinica], 69(5), 666–676. 29063114

[eph13658-bib-0063] Zhang, H. , Zhang‐Sun, Z. Y. , Xue, C. X. , Li, X. Y. , Ren, J. , Jiang, Y. T. , Liu, T. , Yao, H. R. , Zhang, J. , Gou, T. T. , Tian, Y. , Lei, W. R. , & Yang, Y. (2023). CTRP family in diseases associated with inflammation and metabolism: Molecular mechanisms and clinical implication. Acta Pharmacologica Sinica, 44(4), 710–725. 36207402 10.1038/s41401-022-00991-7 PMC10042840

[eph13658-bib-0064] Zhang, N. , Li, P. , Lin, H. , Shuo, T. , Ping, F. , Su, L. , & Chen, G. (2021). IL‐10 ameliorates PM2.5‐induced lung injury by activating the AMPK/SIRT1/PGC‐1α pathway. Environmental Toxicology and Pharmacology, 86, 103659. 33862202 10.1016/j.etap.2021.103659

[eph13658-bib-0065] Zhao, B. , Li, M. , Li, B. , Li, Y. , Shen, Q. , Hou, J. , Wu, Y. , Gu, L. , & Gao, W. (2024). The action mechanism by which C1q/tumor necrosis factor‐related protein‐6 alleviates cerebral ischemia/reperfusion injury in diabetic mice. Neural Regeneration Research, 19(9), 2019–2026. 38227531 10.4103/1673-5374.390951 PMC11040306

[eph13658-bib-0066] Zheng, H. , Huang, S. , Wei, G. , Sun, Y. , Li, C. , Si, X. , Chen, Y. , Tang, Z. , Li, X. , Chen, Y. , Liao, W. , Liao, Y. , & Bin, J. (2022). CircRNA Samd4 induces cardiac repair after myocardial infarction by blocking mitochondria‐derived ROS output. Molecular Therapy, 30(11), 3477–3498. 35791879 10.1016/j.ymthe.2022.06.016 PMC9637749

[eph13658-bib-0067] Zheng, W. F. , Zhang, S. Y. , Ma, H. F. , Chang, X. W. , & Wang, H. (2019). C1qTNF‐related protein‐6 protects against doxorubicin‐induced cardiac injury. Journal of Cellular Biochemistry, 120(6), 10748–10755. 30719766 10.1002/jcb.28366

[eph13658-bib-0068] Zhou, B. , & Tian, R. (2018). Mitochondrial dysfunction in pathophysiology of heart failure. Journal of Clinical Investigation, 128(9), 3716–3726. 30124471 10.1172/JCI120849 PMC6118589

